# Air–water interface of submerged superhydrophobic surfaces imaged by atomic force microscopy

**DOI:** 10.3762/bjnano.8.167

**Published:** 2017-08-11

**Authors:** Markus Moosmann, Thomas Schimmel, Wilhelm Barthlott, Matthias Mail

**Affiliations:** 1 Nees Institute for Biodiversity of Plants, University of Bonn, Venusbergweg 22, D-53115 Bonn, Germany; 2 Institute of Applied Physics and Institute of Nanotechnology, Karlsruhe Institute of Technology (KIT), Hermann-von-Helmholtz-Platz 1, D-76344 Eggenstein-Leopoldshafen, Germany; 3 Institute of Crop Science and Resource Conservation (INRES) – Horticultural Science, University of Bonn, Auf dem Hügel 6, D-53121 Bonn, Germany

**Keywords:** AFM in liquid, air retention, atomic force microscopy, bionics, Salvinia effect

## Abstract

Underwater air retention of superhydrophobic hierarchically structured surfaces is of increasing interest for technical applications. Persistent air layers (the Salvinia effect) are known from biological species, for example, the floating fern *Salvinia* or the backswimmer *Notonecta.* The use of this concept opens up new possibilities for biomimetic technical applications in the fields of drag reduction, antifouling, anticorrosion and under water sensing. Current knowledge regarding the shape of the air–water interface is insufficient, although it plays a crucial role with regards to stability in terms of diffusion and dynamic conditions. Optical methods for imaging the interface have been limited to the micrometer regime. In this work, we utilized a nondynamic and nondestructive atomic force microscopy (AFM) method to image the interface of submerged superhydrophobic structures with nanometer resolution. Up to now, only the interfaces of nanobubbles (acting almost like solids) have been characterized by AFM at these dimensions. In this study, we show for the first time that it is possible to image the air–water interface of submerged hierarchically structured (micro-pillars) surfaces by AFM in contact mode. By scanning with zero resulting force applied, we were able to determine the shape of the interface and thereby the depth of the water penetrating into the underlying structures. This approach is complemented by a second method: the interface was scanned with different applied force loads and the height for zero force was determined by linear regression. These methods open new possibilities for the investigation of air-retaining surfaces, specifically in terms of measuring contact area and in comparing different coatings, and thus will lead to the development of new applications.

## Introduction

**Figure 1:**
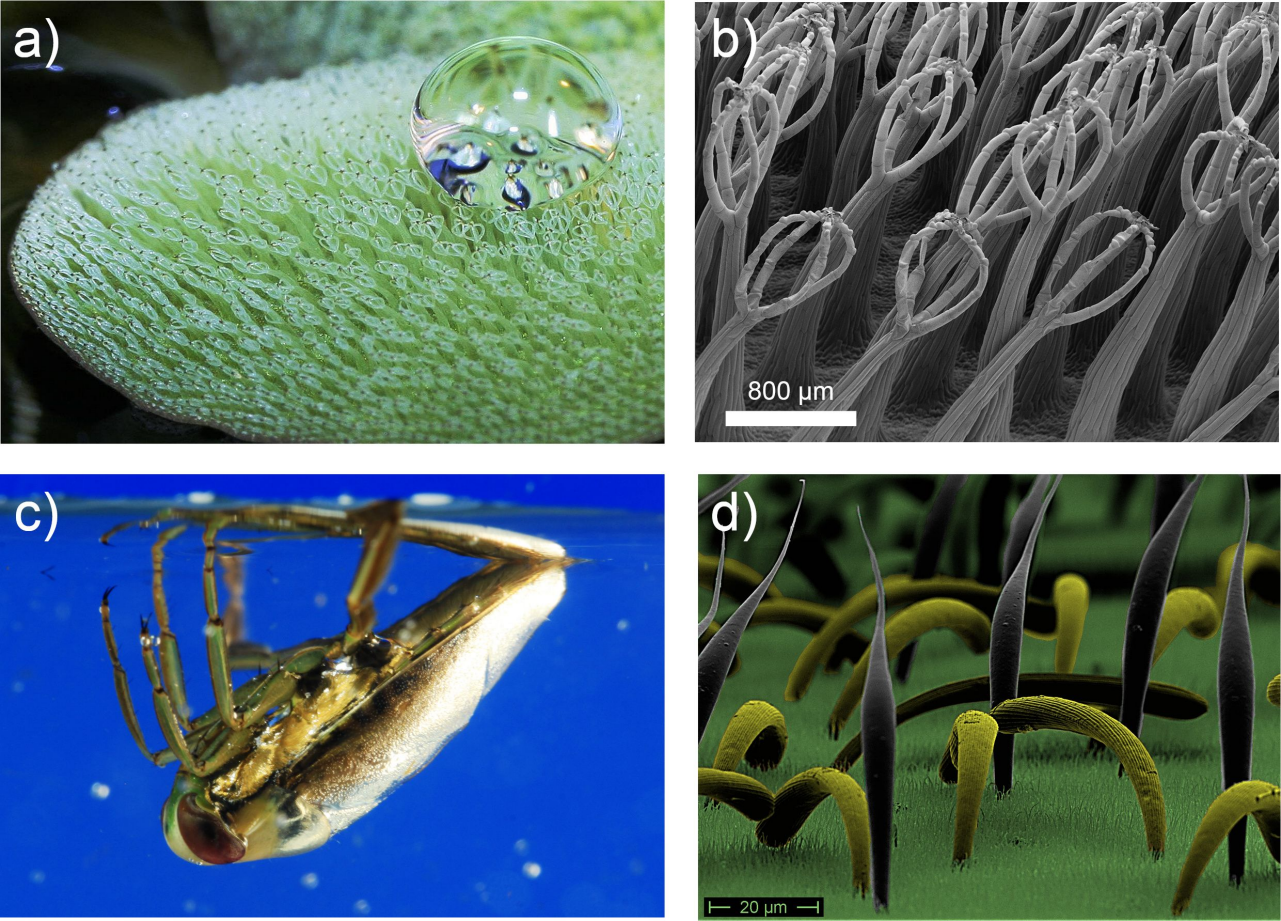
Biological role models of air-retaining Salvinia effect surfaces. a) The floating fern *Salvinia molesta* has one of the most complex surface structures in plants. Reproduced with permission from [5], copyright 2010 Wiley-VCH Verlag GmbH & Co. KGaA. b) With its egg-beater-like trichomes with terminal hydrophilic anchor cells, *Salvinia* is able to maintain air layers for many weeks under water. c) The backswimmer *Notonecta* keeps a persistent air layer on its forewings even when it moves underwater at high velocity. The silvery shine is due to the total internal reflection of light at the air–water interface. d) The backswimmers double structure of longer hairs (grey and yellow) and a dense “carpet” of smaller hairs (microvilli, green) is responsible for the long-term air-retention capability.

Air retention is one of the many fascinating aspects of superhydrophobic surfaces, offering promising new capabilities for technical applications [1]. Starting with the discovery of the lotus effect in 1997 [2], new fields in surface technology have been realized [3–4]. In recent years, the Salvinia effect – the long term stabilization of an air layer on a submerged surface – has gained increasing interest. There is great potential for various technical applications utilizing this effect, for example, drag reduction, antifouling or anticorrosion applications, and underwater sensory systems. Biological surfaces are the basis of the discovery and are models for the development of biomimetic surfaces. The conquest of land some 450 million years ago led to the evolution of an almost endless variety of surface structures and functionalities in plants and animals [3]. One of the most complex plant surfaces is exhibited by the giant floating fern *Salvinia molesta* (Figure 1a,b). With its elastic egg-beater-like shaped trichomes and chemical heterogeneities [5], the fern is capable of maintaining a stable air layer underwater for several weeks. Another example is the backswimmer *Notonecta* (Figure 1c,d) with its double structure of longer hairs and a dense “carpet” of so-called microvilli.

Based on the analysis of hundreds of aquatic and semiaquatic species, four criteria for the maintenance of persistent air layers underwater have been identified [3]. The structures on these biological role models range from the millimeter (e.g., *Salvinia*) to the micrometer (e.g., *Notonecta*) scale.

The shape of the air–water interface is of crucial importance for the diffusion and stability of the air layer under dynamic conditions. Konrad et al. set up a method allowing for the prediction of the stability and the persistence of air layers [6]. Air–water interfaces are very sensitive and vulnerable to almost any kind of disturbance. For this reason, several methods have been developed to allow for imaging of the interface, for example, by using confocal microscopy [7] or freezing technologies [8]. Most of the methods used so far are limited to structures with features in the micrometer range. However, atomic force microscopy (AFM) is a suitable instrument to study smaller dimensions but is still rarely used to image air–water interfaces. The most prominent exception is the investigation of nanobubbles [9–10]. These are bubbles of air forming on immersed hydrophobic substrates with typical diameters of 100 nm to 1 µm and heights of 10–100 nm [11]. Because of their surprising stability [12], they are relatively easy to image in different AFM modes of operation [9,13–14]. Generally, for the characterization of highly compressible surfaces by AFM, dynamic modes are difficult to apply [15–17].

The air–water interface of submerged lotus leaves was analyzed by magnetic alternating current mode [18], where the interface is “little disturbed” according to the authors. But this disturbance could be enough to affect the interface [19].

**Figure 2:**
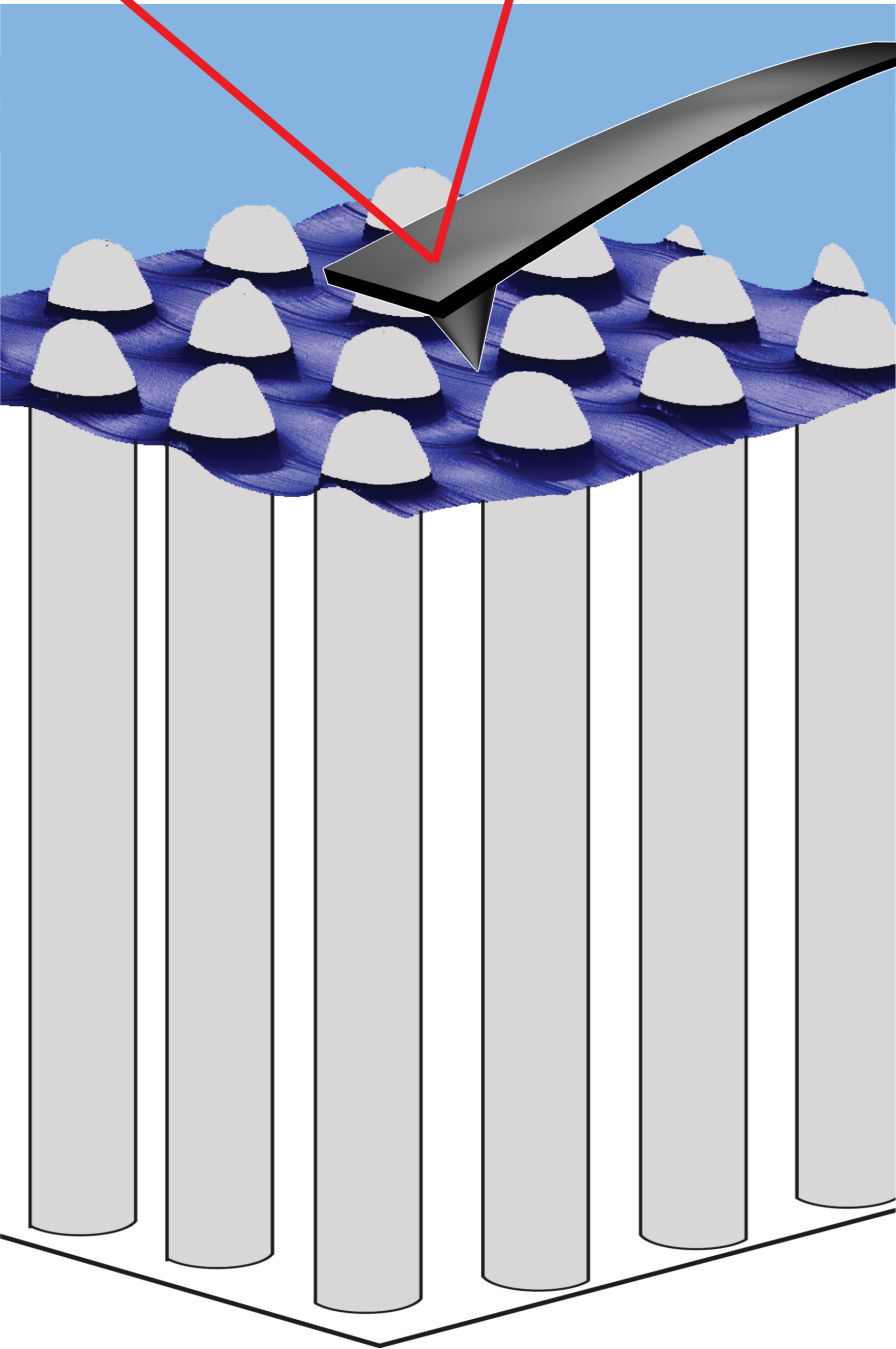
Representation of the measurement of the air–water interface on submerged structures performed in this study. This pictorial image is intended to provide a general overview of the method used in this work, i.e., the proportions of the individual elements are not to scale. The cantilever, for instance, is much larger in comparison to the pillar structure.

Here, we present the first results of imaging the air–water interface of submerged superhydrophobic air-retaining technical surfaces by regular contact mode AFM. We demonstrate the shape of such an interface with unprecedented resolution. A pictorial representation of the measurement is shown in Figure 2. As it is designed to provide a general overview, the proportions of the individual elements are not to scale. We are able to scan without resulting force (set point = 0 nN) to determine the depth of the water layer penetrating into the underlying structured surface. To confirm the results we utilized an additional approach. By scanning with varied applied force, we determined the corresponding depth of water. By linear regression we compute the value for the 0 nN setpoint. The results of both methods were in good agreement. We conclude with a suggested model for the contact between the AFM tip and the interface.

## Results and Discussion

**Figure 3:**
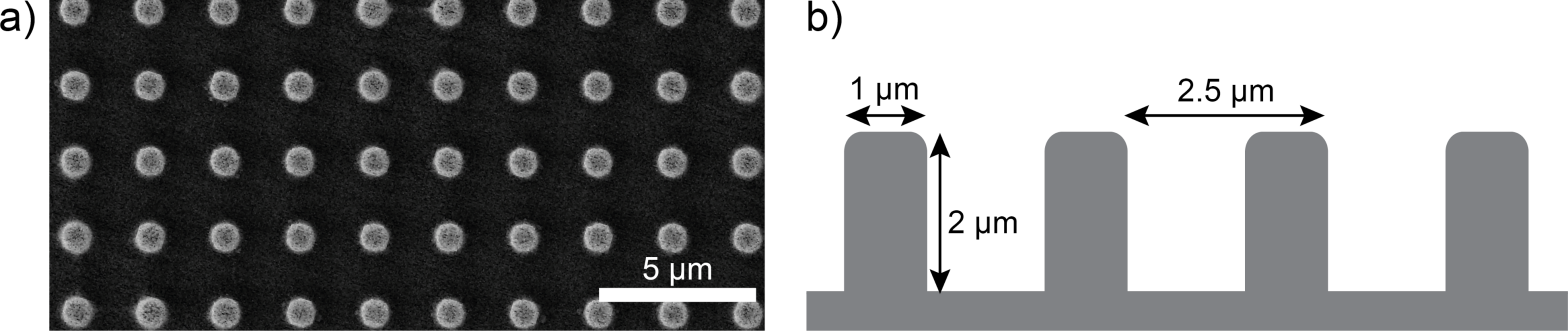
Architecture of the epoxy replica samples used in this study. a) SEM image of a sample with micro-pillars on its surface (top view). b) Schematic cross-section of the periodically ordered micro-pillars on the surface (height 2 µm, diameter 1 µm, pitch 2.5 µm).

This study aims to image and analyze the shape of the air–water interface of air-retaining surfaces by AFM. This goal was achieved using epoxy resin samples with a micro-pillar structure at their surface. The samples were produced in a two-step molding process [20] (see Experimental section) and were based on silicon surfaces with micro-pillars structured by reactive ion etching (RIE). Tegotop^®^ was applied as a superhydrophobic coating. Figure 3a shows an SEM image (top view) of the final epoxy resin sample with micro-pillars on its surface. Figure 3b schematically illustrates the cross-section with dimensions of 1 µm in diameter, 2 µm in height and a pitch of 2.5 µm. Based on a variety of different parameters, these proved to be the best choice for AFM measurements.

**Figure 4:**
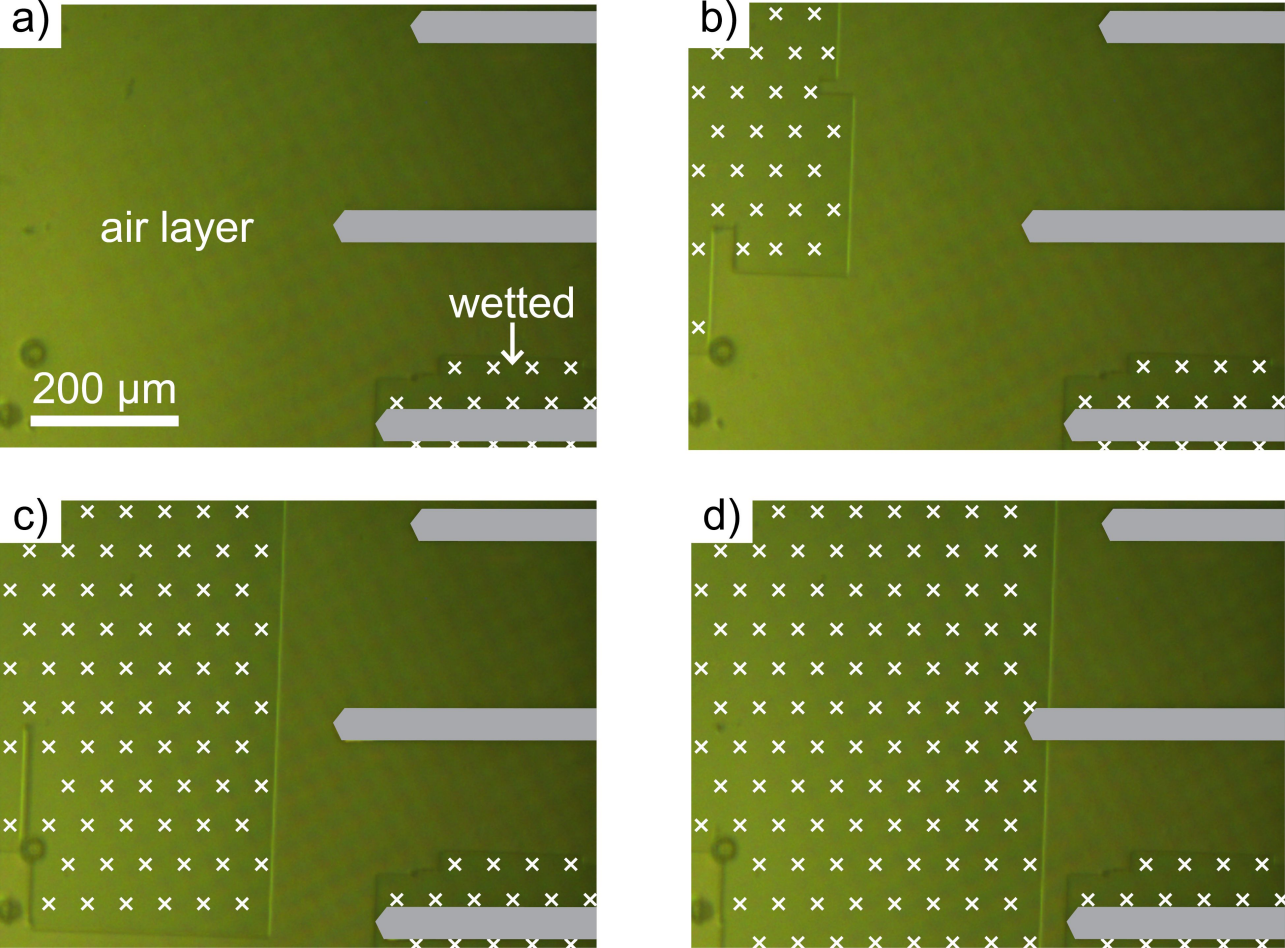
Photographic images of a submerged air-retaining sample with micro-pillars taken over a duration of 15 minutes using the AFM camera. A chip holding three cantilevers (schematically indicated in grey) was installed, as can be seen on the right portion of each image. White crosses indicate areas that have been wetted. a) In the beginning, the surface shows air retention, with the exception of the lower right area. b) After about 3 minutes, a sudden change of the wetting state occurred, as the air layer collapsed on the upper left. c, d) The collapse occurred stepwise and erratically, propagating towards the cantilevers. In all cases, the interfaces separating the wetted areas from the air-retaining areas followed exactly the alignment of the micro-pillars.

During AFM experiments, it is important to know whether a submerged sample shows stable air retention, which can be optically verified. Figure 4 shows a series of four images of a submerged sample taken over a time span of 15 minutes. Figure 4a shows the sample that was placed in the AFM system, as can be seen by the three cantilevers on the right-hand side marked in grey. Below the lower cantilever the sample is already wetted (Wenzel state), as marked with white crosses. The rest of the sample shows air retention (Cassie–Baxter state). Figure 4b was taken after three minutes. In the upper left area, the air layer has collapsed. After a duration of an additional 12 minutes, the wetted area increased (Figure 4c) until it finally reached the middle cantilever (Figure 4d). This wetted area advancement occurred stepwise and erratically. Noticeably, the interface separating the areas of air retention from the wetted areas are aligned orthogonal to each other. The pillars of the sample are aligned in the same direction as the interfaces. Thus the collapse of the air-layer advances line-by-line. There is an additional, faintly visible pattern extending diagonally over the entire image attributed to interference.

**Figure 5:**
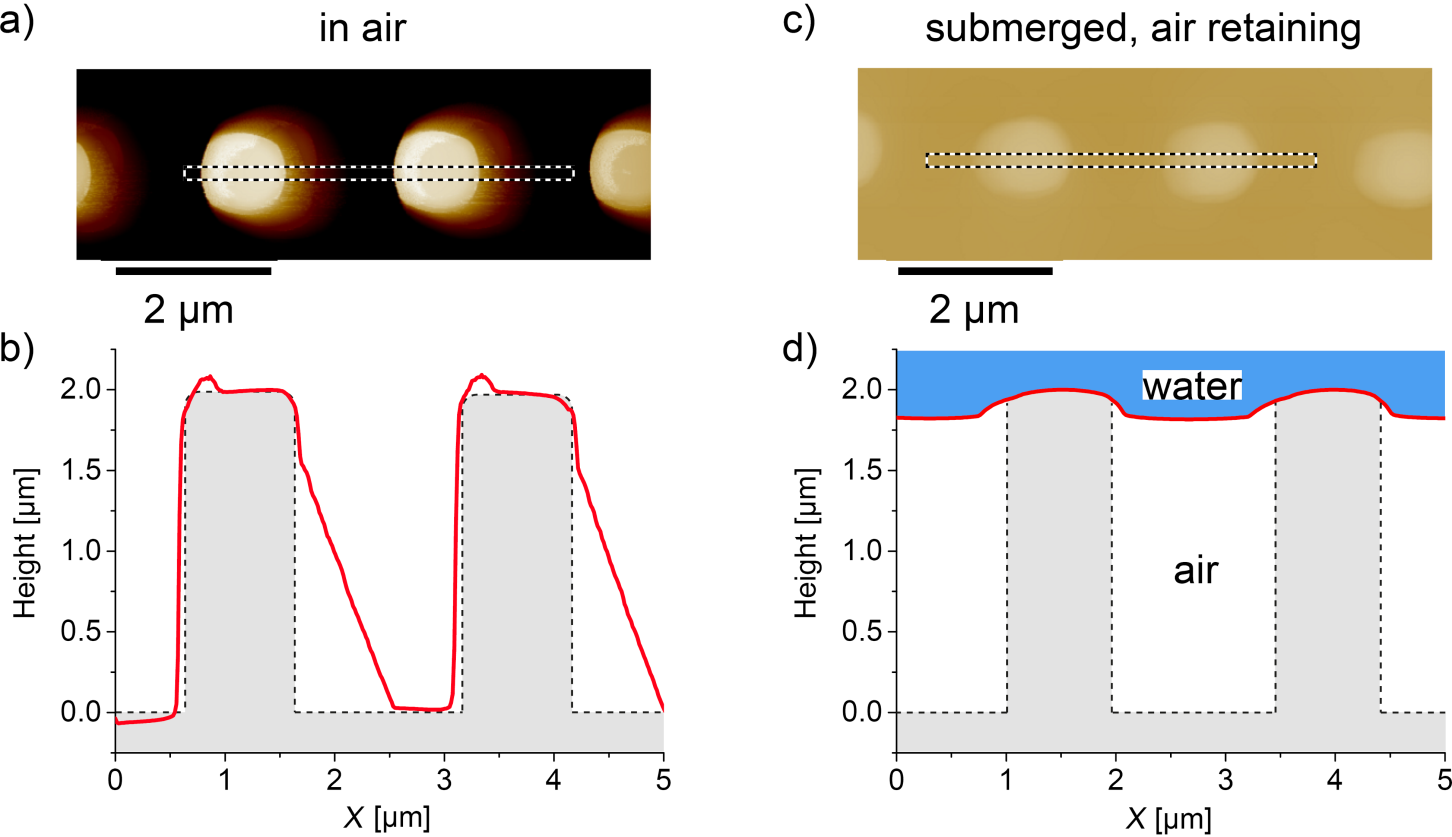
AFM images (a, c) and the corresponding cross-sections (red lines in b, d) of the sample. The image in a) was taken in ambient conditions in tapping mode and confirms the pillar height of 2 µm. The light coloration on the right side of each pillar is an unavoidable artifact in AFM imaging, originating from the pyramidal shape of the AFM tip. This is also displayed by the corresponding cross section (red line) in b). The actual topography is also schematically illustrated (grey). c) AFM image taken in contact mode of a submerged air-retaining sample. d) The red line shows the cross-section with a total height of only 185 nm. Hence the AFM tip does not reach the base of the sample but rather follows the air–water interface, which is 185 nm below the pillar tops. The height of the air layer beneath is 1.815 µm, which translates to at least 90% of the total pillar height.

With samples showing long-term stable air retention, we were able to image the air–water interface by AFM. In Figure 5, the data measured from the ideal sample in ambient conditions and in water are compared. The image of Figure 5a was taken in ambient conditions in tapping mode. It shows a row of pillars validating the data presented in Figure 3. However, the corresponding cross-section (Figure 5b, red line) contains two artifacts: the additional elevation at the pillar top is due to the feedback loop of the AFM system causing an overshoot in the height signal. The slope on the right, which seems to be too flat, is unavoidable as it is caused by the pyramidal shape of the AFM tip. The actual topography of the pillars is schematically drawn in grey. When the sample is submerged, an air layer is maintained and the air–water interface can be imaged by AFM using the nondynamic contact mode (Figure 5c). In this case we used a set point of 6 nN and measured the pillar height to be only 185 nm. This is because the AFM tip did not reach the base of the sample between the pillars but rather scanned the air–water interface. This is shown in Figure 5d with the corresponding cross-section of the measurement (red line). As the depth of the water was 185 nm, the air layer beneath had a height of 1.815 µm. Hence, more than 90% of the lateral surface area of the pillars remained dry.

**Figure 6:**
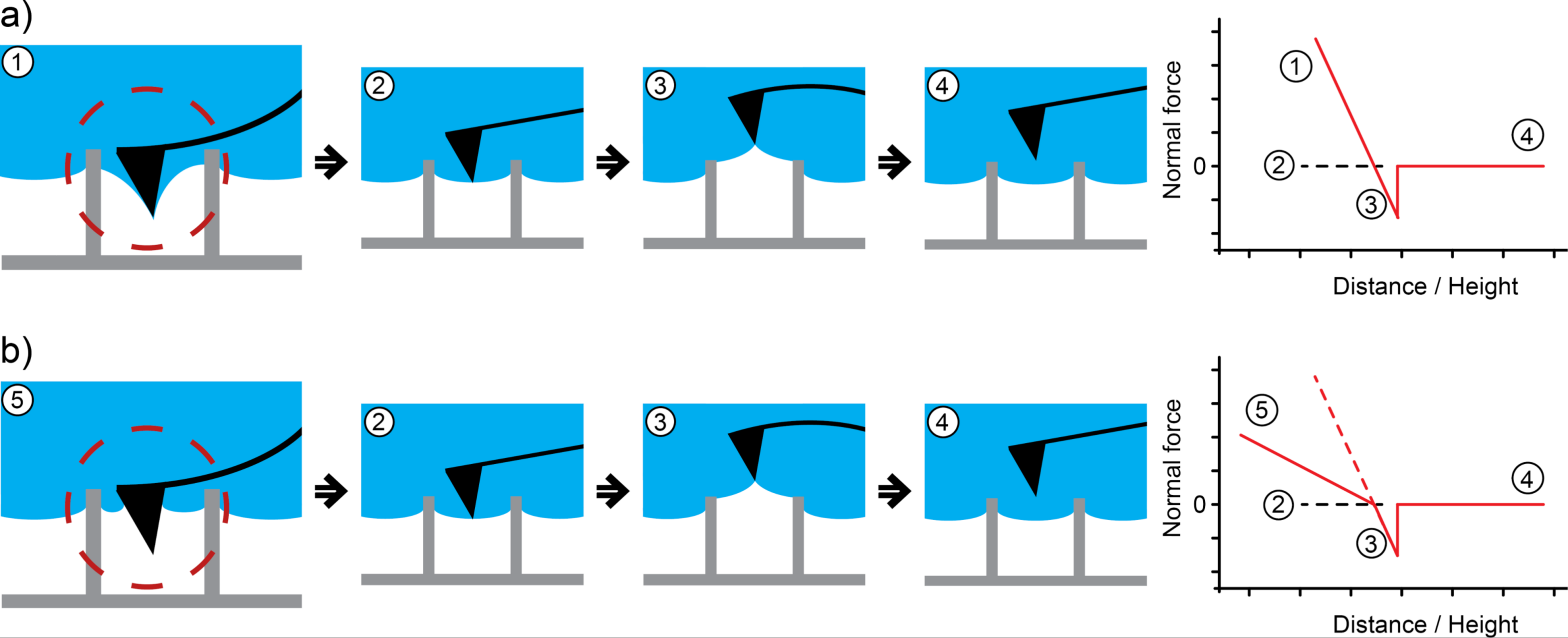
Two possibilities for the contact between AFM tip and air–water interface: a) the interface is pinned to the tip while it is pressed towards the base; b) the tip penetrates the interface. To find the model that best describes our system we consulted force–distance curve measurements like those schematically shown on the right. The individual points in the curves correspond to the images. 1 and 5: The tip is pressed towards the surface. The normal force is positive. 2: No resulting force applied by the tip. 3: The tip is withdrawn from the surface. The restoring force of the air–water interface pulls the tip downwards resulting in a negative force. 4: After the tip loses contact with the interface, the acting force is zero. Subsequently, the force–distance curves should differ in cases a) and b). As shown on the right, we expect the curve to show a change in slope at point 2 in case b).

Under variation of the normal force applied to the interface by the AFM, we measured different penetration depths of the water into the structure. This is not surprising as the air–water interface cannot be considered a solid layer; it behaves instead like a membrane. To provide a reliable interpretation of the AFM data obtained, it is important to understand the contact between the air–water interface and the AFM tip during scanning. In particular, the question arose: what happens when the normal force is increased? Two possible options are presented in Figure 6. In case of a “pinning” situation of the air–water interface to the tip (schematically illustrated in image 1 in Figure 6a), an increase of the normal force will pull the interface down. Another possibility of the contact is illustrated in image 5 (Figure 6b): the AFM tip may penetrate the air–water interface. To determine which of the models is most realistic, we measured force–distance curves. Here the distance of the AFM tip to the surface is varied and the deflection of the cantilever is detected. With the cantilever calibrated, the deflection is automatically translated into the corresponding force. Considering that not only the cantilever but also the air–water interface acts according to Hooke’s law, we expect the pinning behavior to be relevant (as illustrated in Figure 6a). The images 1–4 show specific situations during the force–distance curve measurement, and the diagram furthest to the right illustrates the estimated curve. Image 1 illustrates the AFM tip as it pushes the air–water interface down into the pillar structure towards the base of the sample. Here the normal force applied is maximal. As the AFM tip is retracted, the force on the tip decreases. At point 2, the air–water interface is at equilibrium and no resulting force can be detected. Beyond this point, the further retraction forces the cantilever to buckle due to pinning, and the AFM tip is pulled down by the air–water interface. Hence the measured force is negative (point 3). Supposing the interface behaves Hookean, there should not be any change in the slope of the curve at point 2. After the maximum adhesion is reached, the pinning ends abruptly and the tip is detached from the interface. Subsequently, no resulting force is applied to the cantilever (point 4).

Figure 6b illustrates the situation expected for a penetration of the air–water interface in the case of pressure by the tip. The behavior displayed in the images 2–4 is the same as in Figure 6a. However, if the tip penetrates the air–water interface as shown in image 5, we expect a different behavior in a manner illustrated in the graph on the right side, i.e., to demonstrate a change in slope at point 2.

**Figure 7:**
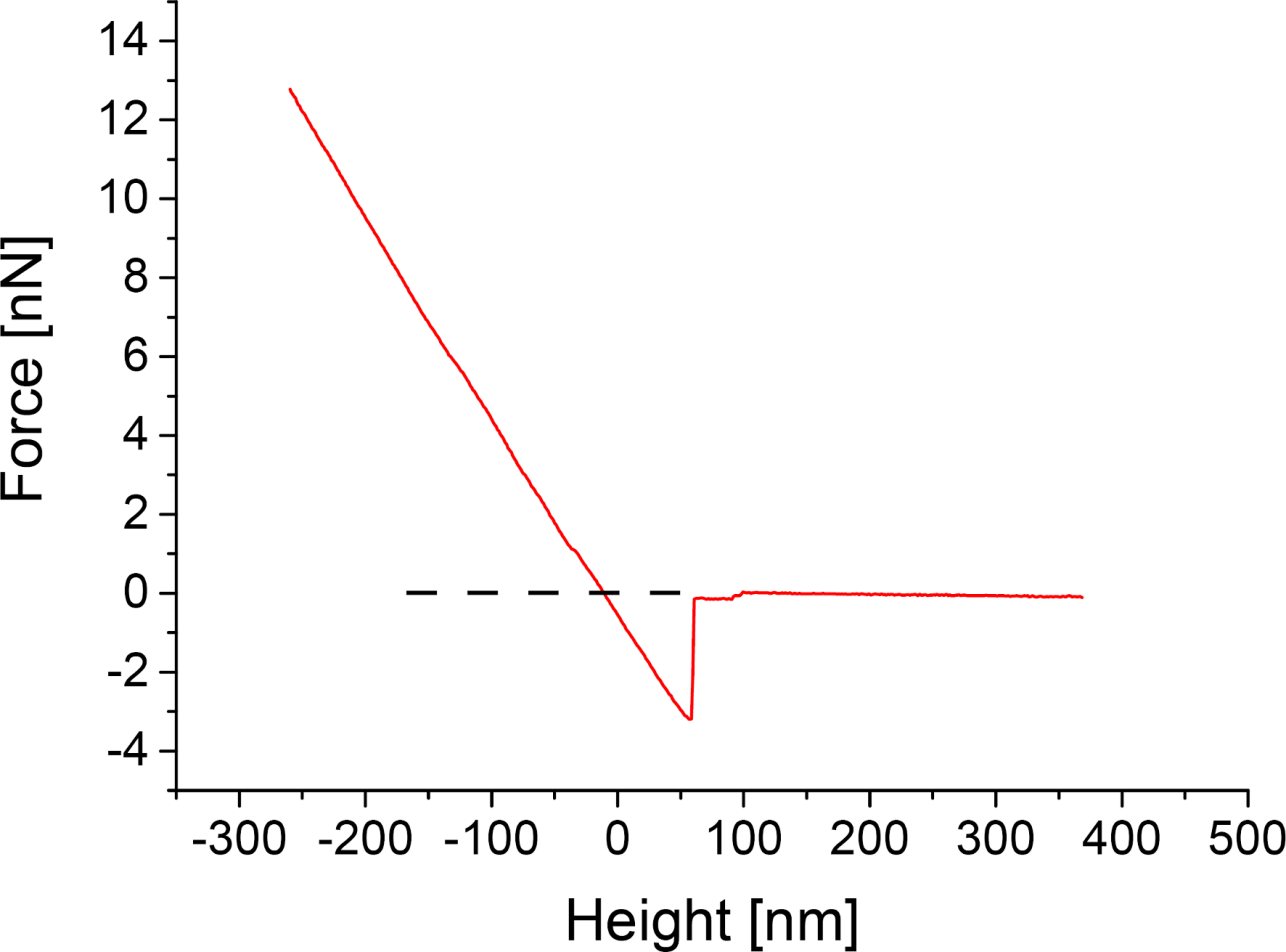
Force–distance curve measured at the air–water interface of a submerged air-retaining sample. Positive height values: the tip is above the air–water interface. The curve shows the characteristics proposed in Figure 5a exhibiting no change in slope between attractive (positive force) and repulsive (negative force) regimes (dashed line, see point 2 in Figure 6). We conclude that the air–water interface is not penetrated by the AFM tip but rather pinned to its apex. We calculated the force constant of the interface to be 0.07 N/m from the gradient of the curve in the repulsive force regime.

The experimental result of such a force–distance curve is shown in Figure 7. It was taken at the air–water interface in the center of a square formed by four pillars. All curves we took looked similar to the one shown here and confirm the situation of pinning we proposed in Figure 6a. Hence the tip pushes the air–water interface down upon advancing to the sample surface. Moreover, the gradient of the force–distance curve in the negative height regime is the cumulative force constant *k* of the cantilever and the air–water interface according to Hooke’s law: *F = k* × height. As we calibrated the AFM cantilever in advance, we were able to determine the force constant of the interface in this case to be 0.07 N/m.

**Figure 8:**
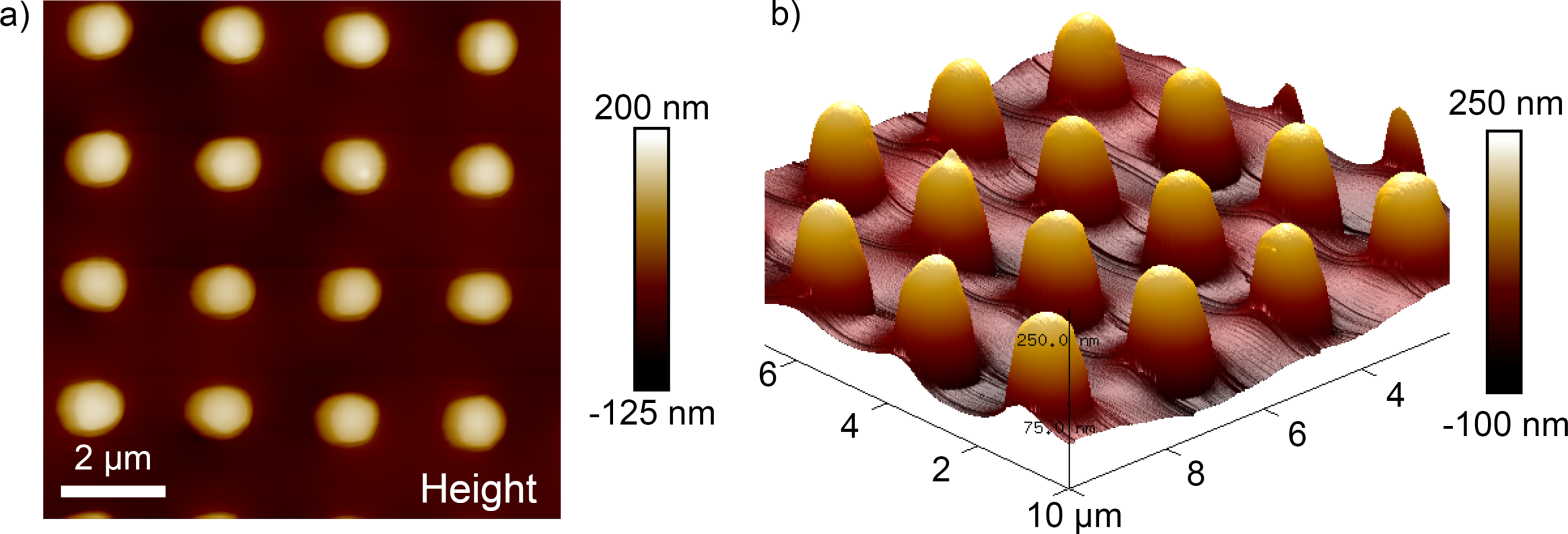
a) AFM image in contact mode taken on a submerged air-retaining sample with an applied force of 6.4 nN. An area of 10 × 10 µm covering 16 pillars (bright spots) was scanned. b) The darker area between the pillars in a) indicates the shape of the air–water interface and can be better seen in the 3D representation of the data. Note that the x–y plane is scaled in micrometers and the height is scaled in nanometers.

In agreement with the data of the force–distance curves taken at the air–water interface, we measured different depths of the water penetrating into the pillar structure for different force loads applied. To analyze the depth, we imaged areas of 10 × 10 µm including 16 individual pillars with different force loads for each image. Figure 8a shows such an image acquired with a force load of 6.4 nN. The air–water interface is not completely flat as can be seen in the 3D representation of the data displayed in Figure 8b. We used an averaging method called “particle analysis” provided by the scan software to determine the water depth. Each pillar emerging from the interface is treated as a single particle and its height in relation to the surrounding background (in this case the air–water interface) is measured.

**Figure 9:**
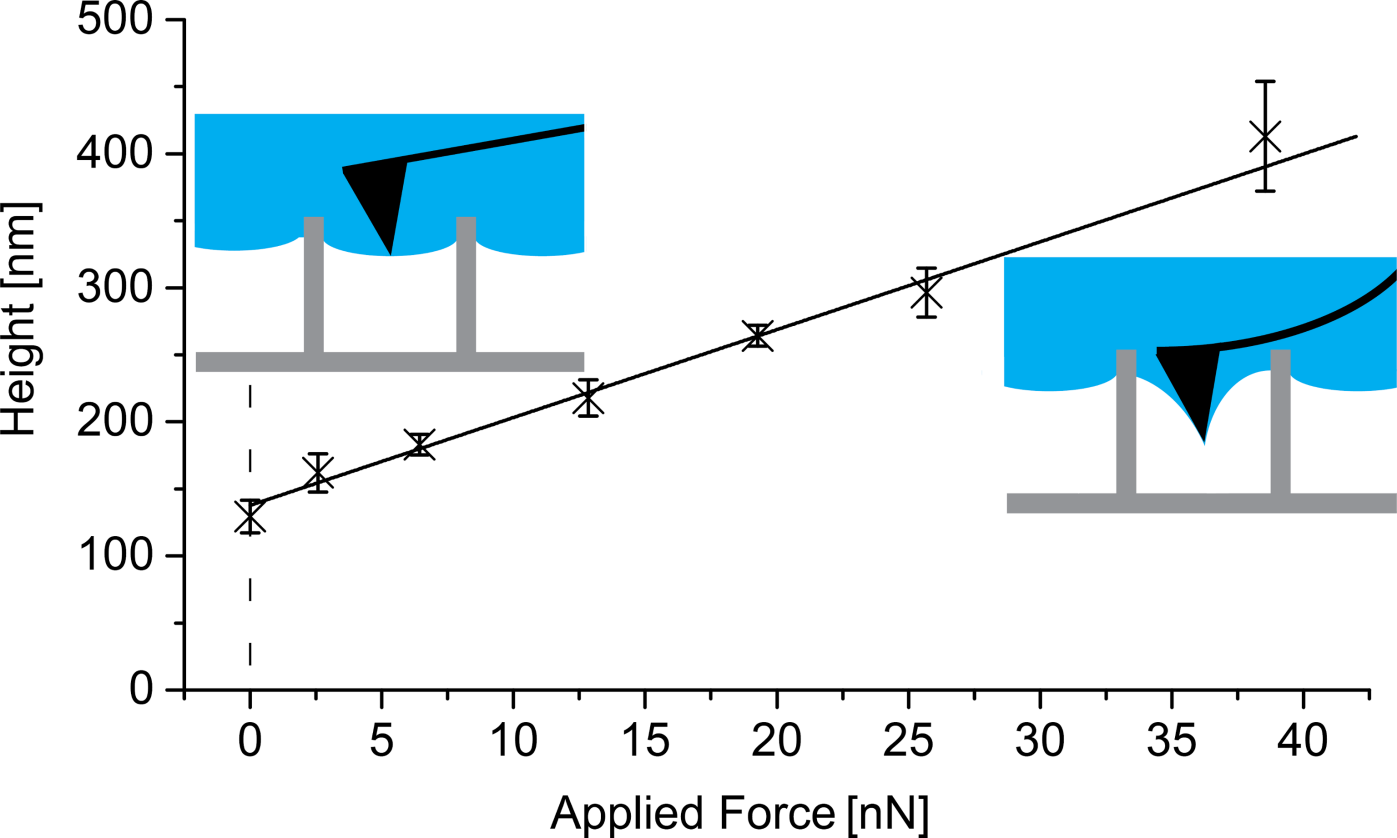
Water depth relative to the pillar tops as a function of force applied during scanning. For each data point, a complete 10 × 10 µm scan (similar to that shown in Figure 8a) was taken with an individual and constant force applied to the air–water interface by the cantilever. The values, as well as the corresponding error bars, were determined by the “particle analysis” procedure of the AFM software. The higher the applied force, the larger the compression of the air and hence distance between the pillar tops and air–water interface, as schematically shown in the insets. With a set point of 0 nN (zero resulting force) a height of 129 ± 12 nm was measured, which is in very good agreement with the value of 138 ± 4 nm determined by the linear regression of all data points.

**Figure 10:**
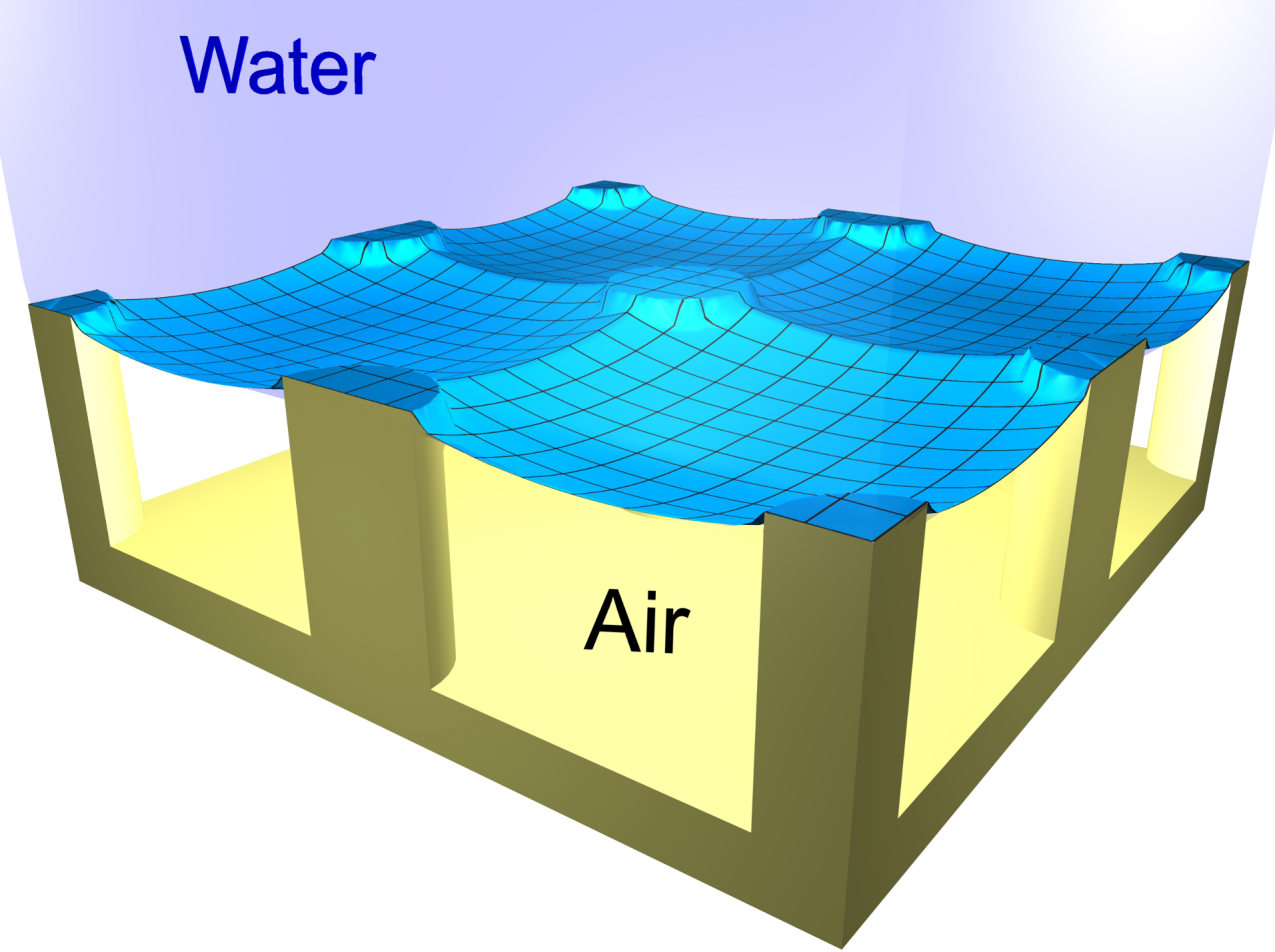
An artistic 3D representation of the air–water interface, which does not represent actual measurement data or experimental dimensions.

Figure 9 shows a set of data points of such an area imaged with different force loads (set point) between 0 and 40 nN. A selection of AFM images taken with set points of 0 nN, 19.3 nN and 38.5 nN is shown in Figure S1 in Supporting Information File 1. The depth of the water shows a linear dependence to the applied force, as the linear regression of the data points fits perfectly within the error bars of the individual points. The higher the force applied, the deeper the water is pushed into the structures. The two schematic insets provide a visual representation of this interpretation. Most important is the data point where no resulting force is applied to the interface (set point 0 nN). To achieve a zero force scan one has to set a positive set point for the approach of the AFM tip and to reduce it to zero after the tip is engaged. The measured depth of 129 ± 12 nm denotes that more than 90% of the total sample surface area remained dry. This means that the height of the air layer is 1.871 µm. Additionally, the actual measurement with a set point of 0 nN matches the best fit straight line of all data points, which returns a value of 138 ± 4 nm. We found both methods to be equally suitable in determining the depth of water penetrating into structures of air-retaining surfaces by means of AFM. Moreover, we were able to determine the scope of the air–water interface by scanning with zero force. A purely artistic 3D illustration, intended for better understanding but not based on the actual experimental setup or results, is shown in Figure 10.

## Conclusion

In this study we present a new approach for AFM imaging the air–water interface of submerged air-retaining surfaces with unprecedented nanometer resolution. Beyond imaging the well-known, almost solid-like behaving nanobubbles, we expand the scope of AFM measurements to a much more fragile system. This was achieved by using a nondynamic but nevertheless (when used carefully) nondestructive AFM contact measurement mode.

Besides precisely mapping the shape of the interface, this method also allows an accurate control of the force applied to the interface. By varying this force we simulated different local pressures and were thus able to determine the penetration depth of the water into the hydrophobic pillar structure. The depth is linearly dependent on the force applied in accordance to Hooke’s law. We measured the depth by applying zero resulting force to the interface. This value was confirmed by linear regression of values obtained by scanning with different forces.

Moreover, by taking force–distance curves, we were able to predict a model for the contact between the AFM tip and air–water interface, indicating that only the apex of the tip contacts the interface during imaging.

The methods presented ultimately expand the portfolio of AFM applications. They allow the analysis of various micrometer-structured air-retaining surfaces with regards to geometry, stability and depth of the maintained air layer. Since biomimetic air-retaining surfaces show a great economic potential, they have gained interest in recent years. The methods applied here, presented for the first time, might be of great interest for the further development of these surfaces, as they provide important insights into understanding the basic principles and the design of optimized biomimetic surfaces.

## Experimental

### Fabrication of the micro-pillar samples

The master for the epoxy resin samples used in this study was a silicon wafer covered with micrometer-scale structures created by reactive ion etching (RIE), which were ordered from the Center of Advanced European Studies and Research (Caesar) in Bonn, Germany. The structures were transferred to epoxy resin by a two-step molding process [21]. In the first step, a negative of the silicon master is generated by creating a mold with a poly(vinyl siloxane) dental wax (President Light Body Gel, ISO 4823, PLB; Coltene Whaldent, Hamburg, Germany). In the second step, this mold was filled with a two-component epoxy resin (Epoxidharz L and Härter S, R&G Faserverbundwerkstoffe GmbH, Waldenbuch, Germany). After curing the epoxy resin, the samples were taken out of the casting mold and dip-coated with Tegotop^®^ 210 (Evonik Industries AG, Essen, Germany) to create a superhydrophobic surface.

### Characterization by atomic force microscopy (AFM)

AFM images were made with a commercial AFM system (Dimension ICON, Bruker) operated by a Nanoscope V controller (Bruker). Imaging under ambient conditions was conducted in tapping mode with NSC 15 cantilevers (MikroMasch) with a nominal force constant of 40 N/m and a nominal resonance frequency of 325 kHz. Submerged samples were measured using a commercial liquid cell (Bruker) filled with demineralized water. Images were taken in contact mode with Pt-coated CSC 37 cantilevers (MikroMasch) with force constants between 0.3 N/m and 0.8 N/m. The spring constant of the individual cantilevers was determined by the software Nanoscope (v. 8.15, Bruker) after taking a force–distance curve on a sapphire substrate to determine the sensitivity and a thermal tune. The normal force in contact mode was calculated by multiplying the force constant by the sensitivity and the relative set point of the photodiode given in Volts.

### Characterization by scanning electron microscopy (SEM)

The structured silicon wafers, in addition to the functionalized epoxy resin samples, were imaged by a LEO360 SEM instrument. In the case of the epoxy resin samples, a 30 nm gold layer was sputter-coated onto the surface to enhance their conductivity.

## Supporting Information

File 1 Additional AFM images.
The supporting information shows AFM images of the air–water interface and the corresponding 3D representations obtained with different set points.
