# Supplementary material for: Air–water interface of submerged superhydrophobic surfaces imaged by atomic force microscopy
Source: Beilstein J Nanotechnol. 2017 Aug 11;8:1671–9. doi: 10.3762/bjnano.8.167 (PMC5564253; doi:10.3762/bjnano.8.167)
Supplement: File 1 — Additional AFM images. The supporting information shows AFM images of the air–water interface and the corresponding 3D representations obtained with different set points. [file Beilstein_J_Nanotechnol-08-1671-s001.pdf]

Supporting Information

for

Air–water interface of submerged superhydrophobic surfaces imaged by atomic force microscopy

Markus Moosmann*[1,2], Thomas Schimmel[2], Wilhelm Barthlott[1], and Matthias Mail[1,3]

Address: [1]Nees Institute for Biodiversity of Plants, University of Bonn, Venusbergweg 22, D-53115 Bonn, Germany, [2]Institute of Applied Physics and Institute of Nanotechnology, Karlsruhe Institute of Technology (KIT), Hermann-von-Helmholtz-Platz 1, D-76344 Eggenstein-Leopoldshafen, Germany and [3]Institute of Crop Science and Resource Conservation (INRES) – Horticultural Science, University of Bonn, Auf dem Hügel 6, D-53121 Bonn, Germany

Email: Markus Moosmann - moosmann@uni-bonn.de

* Corresponding author

Additional AFM Images

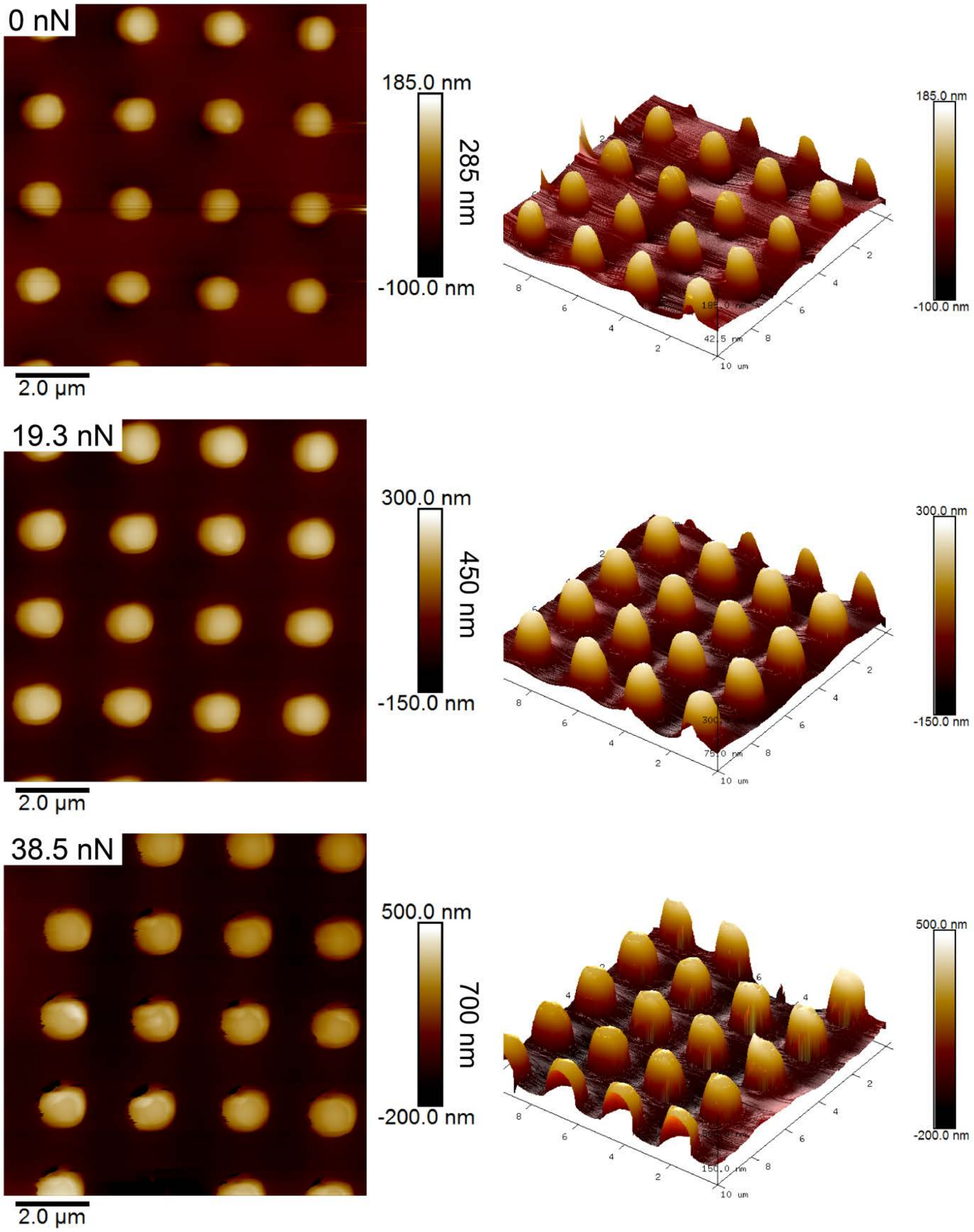

Figure S1: left: AFM height images of submerged pillar sample the taken with different setpoints and corresponding 3D representation (right). Top: 0 nN, middle: 19.3 nN, bottom: 38.8 nN. Note the different height scales.
